# Why is alcohol excluded and opium included in NDPS act, 1985?

**DOI:** 10.4103/0019-5545.33262

**Published:** 2007

**Authors:** Saddichha Sahoo, N. Manjunatha, Baxi Neeraj Prasad Sinha, C. R. J. Khess

**Affiliations:** Central Institute of Psychiatry, Kanke, Ranchi, UK; * Crisis Resolution and Home Treatment, Tees, Esk and Wear Valleys NHS Trust, University Hospital of North Tees, Hardwick Road, Stockton-on-Tees, Cleveland, TS19 8PE, United Kingdom

## THE PROBLEM

Different forms of alcohol have been used in various human societies at least since the beginning of recorded history. As part of the contemporary dynamic of globalization, there has also been an increased use of drugs, which has now reached mammoth proportions. The use of both licit drugs such as alcohol and tobacco and illicit drugs such as cannabis, cocaine and opioids has been acknowledged to have multiple consequences to health, society and economy. According to estimates made by the World Health Report,[1] at least ten thousand million people throughout the world regularly use alcohol and about 13.5 million people use opioids. In India too, the problem is slowly increasing and it is estimated that 75 million people are alcohol users and nearly three million are opioid users, of which a majority require medical treatment and rehabilitation.[2]

A number of other psychoactive substances are being added daily to the present list of psychotropic substances. The entire issue is complex and multifaceted requiring both health measures and efforts to control trafficking / smuggling and manufacture of these drugs. There is a need for the reduction in the demand of drugs of addiction, both legal and illegal, which may otherwise lead to numerous health, family and societal consequences. To combat this, the Government of India formulated the Narcotic Drugs and Psychotropic Substances (NDPS) Act, 1985,[3] which provides the current framework for drug abuse control and sale in this country. Essentially, the Act deals with supply reduction activities of psychotropic substances namely, cannabis, cocaine and opium. However, the absence of alcohol in the list of psychotropic substances is surprising given the fact that mental health professionals consider alcohol to be a psychoactive substance leading to various social, legal, economic and medical complications ranging from gastritis to withdrawal seizures and delirium tremens.[4]

## THE REASONS

The reasons for not including alcohol in the NDPS Act are many, the important ones being (a) prevailing social acceptance even for frequent self-induced intoxication;[5] (b) the high revenues earned by the Government on the sale of alcoholic beverages; (c) prevalence of illicit and locally brewed undistilled forms of alcohol is very high in society and (d) there may be differences in the clinical course of alcohol dependence contrary to other drugs like opium which have been included in NDPS. The last reason is what can be corrected through systematic clinical studies, which have not been conducted until now.

## THE SOLUTION

### Our study

We therefore aimed at evaluating the reasons for inclusion of opium and exclusion of alcohol from NDPS by comparing and contrasting the course of dependence for both substances. We recruited consecutively admitted patients of ≥ 18 years of age for treatment of dependence in our Centre for Addiction Psychiatry, Central Institute of Psychiatry, Ranchi, India with International Classification of Diseases-10-Diagnostic criteria for research (ICD-10-DCR) diagnosis of alcohol dependence syndrome or opioid dependence syndrome and obtained their written informed consent. We excluded other comorbid psychiatric disorders, substance dependence or general medical conditions requiring additional treatment. We administered the SSAGA-II[6] (Semi-Structured Assessment for the Genetics of Alcoholism-II) test to all our subjects after detoxification.

SSAGA II is a poly-diagnostic instrument, which was designed to assess the physical, psychological and social manifestations (in other words, in terms of the criteria of ICD-10-DCR dependence). After the interview, data was transferred to the ICD-10 tally sheets of the respective alcohol or opioid sections of SSAGA-II. We considered the earliest age of any items of respective criteria of ICD-10-DCR dependence which was taken as the age of first onset of respective criteria of dependence. We also considered the age of onset of ICD-10 dependence as the age of first onset of the third criterion among the six criteria.

### Our findings

We found that the total sample size was 112, of which 81 (72%) were alcohol-dependent and 31 (28%) were opioid-dependent. The mean age of the alcohol users was 35.16 ± 10.2 years as compared to the opioid users, whose mean age was 26.09 ± 5.65. This difference was found to be of high statistical significance (*P* < 0.001) and similarly reported in other studies.[7,8]

The mean age at onset of alcohol use in this study was 18.72 years (standard deviation, SD = 6.84) as compared to opioid use being initiated at 20.73 years (SD = 3.93), which was a statistically significant difference (*P* = 0.05). Age at onset of ICD-10-DCR dependence was 27.51 years (SD-9.28) in alcohol users compared to it being 22.05 years (SD = 3.98) in opioid users. Average duration from onset of substance use and from onset of the first criterion of the development of dependence was 8.78 years (SD = 6.7) and 3.17 years (SD = 3.23) respectively in alcohol users whereas it was 1.32 years (SD = 0.89) and 0.65 (SD = 0.56) years respectively. Both were highly significant across both mean durations (*P* < 0.001).

### Our opinion

We believe that ages of onset of both alcohol and opioid use continue to follow a downward trend in that more and more adolescents are beginning to take up substances at an earlier age due to increased media promotion or peer pressure. Earlier studies have noted ages of initiation of alcohol use to be between 20-25 years[8–11] and that of opioid use to be between 23-30 years.[7,12] Alcohol use starts at a relatively earlier age than opioid use (18 vs 20 years) and there is a rapid progress from onset to dependence within just 8.78 years in alcohol-dependent subjects.

There is an even faster progression from the appearance of the first criterion for dependence in alcohol users within just 3.17 years. This rapid downhill course seen in the alcohol group along with the fact that excessive or hazardous use of alcohol has known medical, psychiatric and economic consequences,[13] is a matter of grave concern. Unfortunately, it is widely accepted as a legal consumable drug for adults in many countries including India. However, in our country and culture, the use of alcohol is also deeply woven into the cultural fabric such that it is neither acknowledged as a drug nor is it considered a problem.[5]

Alcohol use is also not included in the NDPS Act, 1985,[14] which is unacceptable as it has been shown that the presence of strict controls and prohibition imposed in many places in the early 20^th^ century were associated with lower levels of consumption and rates of alcohol-related problems, which rose substantially on relaxation of these controls.[15] We therefore feel that there is a strong justification for the health-related professions to step up their health advocacy with respect to policies to reduce rates of alcohol problems.

The crucial need, from a public health perspective, is to consider some legal control especially for the hazardous (or predependent stages) use of alcohol, a recommendation suggested even by the World Health Organization.[16] Alternatively, it may be considered for inclusion into the NDPS Act, 1985. On the other hand, we found that the transition from opioid use to dependence is just 1.32 years along with a dire prognosis of a 2% mortality risk every year and a high mortality rate of about 50%.[17] This coupled with the fact that the “small quantity” clause in NDPS is being misused to divert medical opioids into the open market,[18] we feel that opioid use should be totally banned from society with the exception of medical and research use. It is imperative that there be a regular means of coordination between different levels of government at both the national and subnational levels to monitor the use and regulation of the market.

## CONCLUSION

Considering the comparatively rapid progression to dependence once the first criterion appears and inspite of societal acceptance and arguments in favor of cardio-protective effect of moderate doses of alcohol, we feel that some legal controls should be debated / considered. This should be considered especially for the hazardous (or predependent stages) use of alcohol, which should be included in the NDPS Act. Similarly, use of opioids, which are considered as “hard drugs”, having a dire prognosis and a faster progression of criteria of dependence needs to be totally eradicated from society with a view to protect the future generations.
